# Study on Urban Heat Island Intensity Level Identification Based on an Improved Restricted Boltzmann Machine

**DOI:** 10.3390/ijerph15020186

**Published:** 2018-01-23

**Authors:** Yang Zhang, Ping Jiang, Hongyan Zhang, Peng Cheng

**Affiliations:** 1 College of Urban Economics and Public Administration, Capital University of Economics and Business, Beijing 100070, China; geozhangyang@yeah.net; 2 College of Resources and Environmental Science, Wuhan University, Wuhan 430079, China; 3 State Key Laboratory of Information Engineering in Surveying, Mapping and Remote Sensing, Wuhan 430079, China; zhanghongyan@whu.edu.cn; 4 College of Public Administration, Guangxi University, Nanning 530004, China; rsandgis@yeah.net

**Keywords:** improved restricted Boltzmann machine, urban heat island, intensity level identification, green island, Wuhan, China

## Abstract

Thermal infrared remote sensing has become one of the main technology methods used for urban heat island research. When applying urban land surface temperature inversion of the thermal infrared band, problems with intensity level division arise because the method is subjective. However, this method is one of the few that performs heat island intensity level identification. This paper will build an intensity level identifier for an urban heat island, by using weak supervision and thought-based learning in an improved, restricted Boltzmann machine (RBM) model. The identifier automatically initializes the annotation and optimizes the model parameters sequentially until the target identifier is completed. The algorithm needs very little information about the weak labeling of the target training sample and generates an urban heat island intensity spatial distribution map. This study can provide reliable decision-making support for urban ecological planning and effective protection of urban ecological security. The experimental results showed the following: (1) The heat island effect in Wuhan is existent and intense. Heat island areas are widely distributed. The largest heat island area is in Wuhan, followed by the sub-green island. The total area encompassed by heat island and strong island levels accounts for 54.16% of the land in Wuhan. (2) Partially based on improved RBM identification, this method meets the research demands of determining the spatial distribution characteristics of the internal heat island effect; its identification accuracy is superior to that of comparable methods.

## 1. Introduction

Currently, the world is experiencing rapid and high-intensity urbanization. Along with the creation of the enormous social and economic benefits that urbanization brings, urbanization also profoundly changes city, regional and even global ecological environments [1,2,3,4]. The phenomenon known as urban heat island is one of the worst manifestations of urban ecological problems [5,6,7,8,9]. Howard L introduced the heat island effect in the London climate book in 1833, and the study of the urban heat island effect has interested scholars around the world over the last 200 years [10,11,12,13,14,15]. In recent years, remote sensing information technology has developed rapidly. With the advantages of time synchronization, wide monitoring and continuous detection, thermal infrared remote sensing has become one of the main technical methods for urban ecological and environmental monitoring [16,17,18].

The study of urban heat islands focuses on the distribution of spatial changes in the urban land surface temperature. Urban heat island intensity is a relative-temperature data concept, in opposition to the absolute temperature standard which is strictly divided by the absolute value of the temperature. In the current study on heat island intensity, there are many kinds of calculation methods to determine heat island intensity, which can be mainly summarized as four kinds. The first method defines the difference between the average temperature of the urban suburbs and the average temperature of the city center as the heat island intensity [19,20,21,22]. This method cannot resolve the spatial distribution of urban high-temperature zones. The second method defines the difference between the typical temperature of the urban suburbs and the typical temperature of the highest zones of the city center as the heat island intensity. The typical temperature values of the suburbs needed by this method are difficult to obtain. Regarding these two methods for the calculation of the heat island intensity, they are subject to data and observation conditions, the observation results are different, comparability is poor, and the calculations have much uncertainty. The third method uses the difference between the urban temperature and a reference green space as the heat island intensity [23,24], but this method also has problems, such as the method’s computational complexity and the complicated background support needed for the large-scale urban heat island intensity level identification. The fourth method is the comparative light temperature method, which is divided by the intensity level to give the urban heat island effect. This method began with the work of Sun [25]. They derived the comparative light temperature method based on metaphorical criteria, which were proposed in the remote sensing information model by Ainai [26]. However, this method did not carry out any land surface temperature inversions, and the applicable conditions rely on the surface light temperature. In the division of the heat island intensity level, the majority of studies adopted the method of equal space based on a certain principle [27,28,29]. The disadvantage of this approach is the subjectivity when determining the best split point. The different division points divide the urban heat island structure, resulting in different spatial distributions and variation patterns, which are bound to cause some uncertainty in the research and analysis.

In light of this, this paper proposes a method that identifies the urban heat island intensity level based on an improved RBM (Restricted Boltzmann machine) model. The urban heat island intensity level identifier is obtained by applying a weak supervised learning technique. The identifier automatically initializes the annotation and optimizes the model parameters sequentially until the target identifier is completed, with very little information about the weak labeling of the target training sample, and an urban heat island intensity spatial distribution map is generated.

## 2. Research Area and Data

### 2.1. Research Area

**Figure 1:**
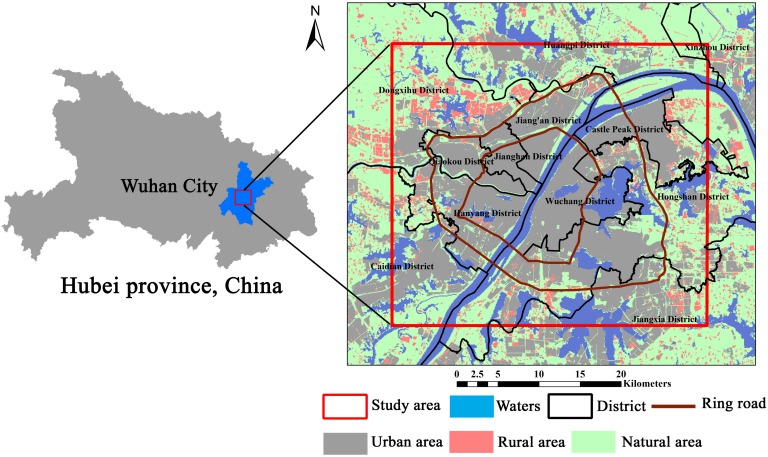
Map of study area.

Wuhan is the central city of the central region and the core city of the middle reaches of the Yangtze River city group. Wuhan is located in the central region of China, with a geographic location in east longitude of 113°41′–115°05′ and north latitude of 29°58′–31°22′. The four boundaries of Wuhan form the shape of a butterfly, from west to east, in the spatial distribution model. The Yangtze River flows through Wuhan and intersects here with its largest tributary, the Hanjiang River. Wuhan is divided into the three parts—Hanyang, Hankou and Wuchang—which are commonly known as the “Wuhan three towns”, by the two rivers. Most of the terrain in Wuhan is dominated by plains; in the middle of Wuhan, residual hills are scattered from east to west, and there are many lakes and pond weirs in Wuhan. Wuhan is known as the “Hundred Lake City”, with more than 166 existing large and small lakes, of which 43 lakes are in the central city. The areas of water in Wuhan, up to 2217.6 km^2^, account for more than one-quarter of the total area of Wuhan, of which the lake area is 803.17 km^2^; the ratio of lake water is ranked first among the major cities of China. Wuhan is a privileged center in terms of China’s economic geography; water and land transportation conditions are very developed. “The centre of nine provinces” reputation has existed since the Qing Dynasty. The elevation of the study area is shown in Figure 1.

### 2.2. Data

Because Landsat/TM (Thematic Mapper), ETM+ (Enhanced Thematic Mapper) and TIRS (Thermal infrared scanner) image data have the advantages of high spatial resolution and easily obtain rich archive time series and data, they have been widely used in land surface temperature inversion of urban heat islands. Therefore, Landsat 8 satellite TIRS image data were selected for this study’s data; the information related to the image acquisition is shown in Table 1. The collected remote sensing images of the study area have no cloudiness, which eliminates the error caused by the cloud cover factor in land surface temperature inversion.

## 3. Research Methods

### 3.1. Technical Route

**Figure 2:**
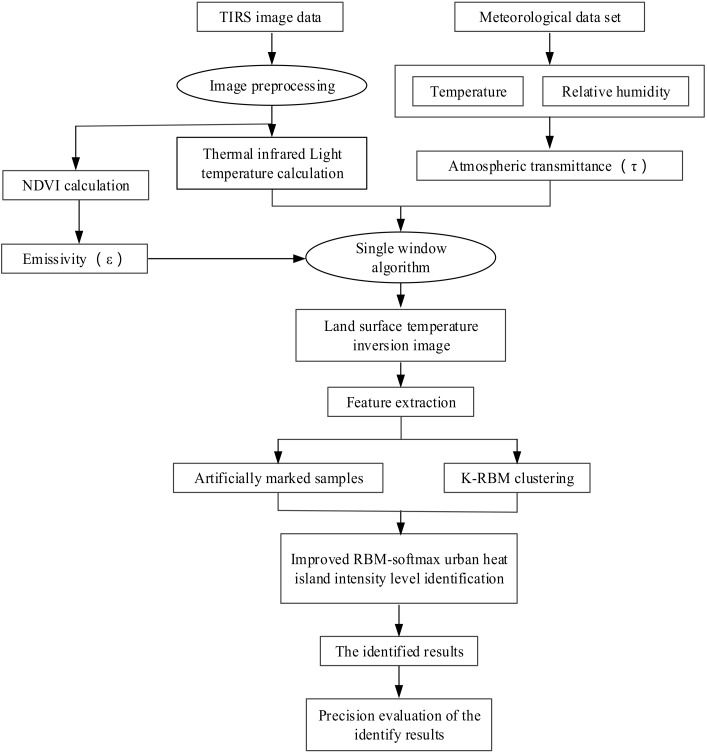
Technical route. RBM: restricted Boltzmann machine; NDVI: normalized vegetation index.

The core steps of urban heat island intensity level identification include land surface temperature inversion, K-RBM clustering (K means clustering of RBM), improved RBM, and reconstruction of urban heat island intensity levels based on the improved RBM. First, the image is pre-processed with the 2016 TIRS data images (23 July 2016, Goddard Space Flight Center, Greenbelt, MD, USA), with the Landsat 8 satellite (National Aeronautics and Space Administration, Washington, DC, USA) as the data source. The projection mode is the Universal Mercator Projection, WGS (World Geodetic System) 1984/UTM (Universal Transverse Mercator) Zone 49N, and the resolution resampling is 30 m by cubic convolution method. Then, in accordance with Rozenstein [30], the single window algorithm can be used for urban land surface temperature inversion. After land surface temperature inversion, K-RBM clustering is performed. The samples with a high confidence level are marked in the clustering result. Based on the principle of minimum error, the most valuable sample set is composed of samples with an artificial mark, which were entered into the improved RBM-softmax urban heat island intensity level identification method. During training, the model parameters are determined by the steepest descent method until convergence, thus finally obtaining the identification results, and the accuracy of the classification is calculated. The core flow of the urban heat island intensity level identification process is shown in Figure 2.

### 3.2. Land Surface Temperature Inversion

The thermal infrared band of the TIRS remote sensing image of Wuhan is introduced into ENVI software (5.3, Harris Corporation, Melbourne, FL, USA). This is the basic data source for urban heat island analysis. While using ArcGIS software (10.2, Environmental Systems Research Institute, Redlands, CA, USA) to convert the required vector data to raster data, a basic image database is established, in a unified format, using the ENVI remote sensing software.

This study aims to quantitatively evaluate the spatial and temporal patterns and changes of the urban heat island. Therefore, surface temperature is the core indicator for this urban heat island research. In addition, Landsat, ETM+ and TIRS remote sensing images are used as data sources, and the inversion of the land surface temperature is performed using a single window algorithm.

(1) Calculate the radiation light temperature

Using the Landsat8 data users handbook provided by NASA (National Aeronautics and Space Administration) landsat science official website to get the model for calculating the radiation brightness temperature [31]:

(1) $$L_{\lambda }=Gains\times \mathrm{DN}+Biases$$

(2) $$T=K_{2}/\ln(K_{1}/L_{\lambda }+1)$$

where *L_λ_* is the radiation intensity received by the TM remote sensor. Gains is the gain factor [unit: (W·m^−2^·sr^−1^·μm^−1^)/DN]; DN is the digital number. Biases is the offset coefficient (unit: W·m^−2^·sr^−1^·μm^−1^). *T* is the radiation light temperature (unit: K); *K*_1_ and *K*_2_ are preset constants. *L_λ_* is the luminance value of the unit spectral range (unit: mW·cm^−2^·sr^−1^·μm^−1^). The values of *K*_1_ and *K*_2_ of band 10 of Landsat8 TIRS are 774.89 and 1321.08.

(2) Calculate the specific emissivity

The specific emissivity of object *ε* is a manifestation of the ability of the object to emit electromagnetic radiation, which is a key parameter in surface temperature inversion. The calculation of emissivity is based on the empirical emissivity ration formula proposed by Van de Griend et al. [32].

(3) ε=1.009+0.047ln(NDVI)

where *NDVI* is the normalized vegetation index [33], *NDVI* > 0 or *ε* = 0.

(3) Land surface temperature inversion

Real surface temperature inversion, using the single window algorithm model, was proposed by Rozenstein [30]. The inversion model is:

(4) $$T_{s}=A_{0}+A_{1}T_{10}-A_{2}T_{11}$$

where LST is the land surface temperature (unit: K), *T*_10_ and *T*_11_ are the radiation light temperatures (unit: K), *ε* is the emissivity of the object, and *τ* is the atmospheric transmittance. The specific calculation process is as follows:

(5) $$\begin{array}{l} A_{0}=a_{10}E_{1}-a_{11}E_{2}\\ A_{1}=1+A+b_{10}E_{1}\\ A_{2}=A+b_{11}E_{2}\\ E_{1}=D_{11}(1-C_{10}-D_{10})/E_{0}\\ E_{2}=D_{10}(1-C_{11}-D_{11})/E_{0}\\ A=D_{10}/E_{0}\\ E_{0}=D_{11}C_{10}-D_{10}C_{11} \end{array}$$

where *C*_10_, *C*_11_, *D*_10_ and *D*_11_ are calculated as follows:

(6) $$\begin{array}{l} C_{i}=\varepsilon_{i}\tau_{i}\\ D_{i}=[1-\tau_{i}][1+(1-\varepsilon_{i})\tau_{i}] \end{array}$$

where the values of *a_i_*, *b_i_* and *τ_i_* are shown in Table 2 and Table 3. We can get these parameters in [30].

### 3.3. K-RBM Clustering

According to the technical route, K-RBM clustering is required before the urban heat island intensity level is identified. The clustering algorithm is as follows:

(1) Initialize K-RBMs, and initialize the rate of error change (EC = 15) of the sum of the reconstruction errors of the eigenvectors of all the pixels in the sample. Initialize all elements in the class chart *Y* to 0, *Y* = {*y*_*m*,*n*_|1 ≤ *m* ≤ *M*, 1 ≤ *n* ≤ *N*}; *y*_*m*,*n*_ indicates the class of any pixel (*m*, *n*) in the sample. The number of the subscript *k* in the 3 × 3 neighborhood of (*m*, *n*) in the initialized graph *Y* is *N*3_*m*,*n*_(*k*) = 0.
(2) Input the feature vectors of all the pixels (A) into the K-RBMs.
  The feature vectors of all the pixels (*m*, *n*) are input into the K-RBMs, and the reconstruction error ($E_{mn}^{k}$) of the K-RBMs for *F_m,n_* is calculated. Based on the principle of minimum error, the extracted feature vector (*F_m,n_*) is classified into K groups (*S_k_*).
  (7) $$F_{m,n}\in S_{k}=\left\{F_{m,n}|k=\arg\min E_{m,n}^{k}\right\}$$
(3) Correct the error of the preliminary classification results. In this step, the mean value (*P_k_*) of the eigenvector (*F_m,n_* ) in *S_k_* is calculated; then, the distance ($D_{m,n}^{k}$) between *F_m,n_* and the cluster center is calculated.
  (8) $$D_{m,n}^{k}={\left\|F_{m,n}-P_{k}\right\|}_{2}+\beta N3_{m,n}(k)$$
  $$\beta =\left\{ \begin{array}{ll} 0 & if\;Q>T1\\ 0.4 & else \end{array}\right.$$
  where *T*1 represents the threshold.

Based on the principle of error minimization, the feature vector (*F_m,n_*) is divided into K groups (*G_k_*). The sample class chart *Y* is updated according to the *k* class of the corresponding pixel of *F_m,n_* in *G_k_*.

(9) $$F_{m,n}\in G_{k}=\left\{F_{m,n}|k=\arg\;\min\;E_{m,n}^{k}\right\}$$

(4) Train the K-RBMs by the feature vector (*F_m,n_*) in *G_k_*. If the algorithm converges, the training is terminated, otherwise perform step 2.

### 3.4. Improved Restricted Boltzmann Machine

The restricted Boltzmann machine is a bipartite graph with two layers of structure. The hidden unit of the RBM models the distribution, based on the visible unit. There is no connection within the layer, and the layers are fully connected. Hinton [34] developed the basic RBM model as a tectonic depth belief network, which brought about widespread concern. Because the RBM units are random, the restricted Boltzmann machine can also be regarded as a random neural network. This model requires strong computing power and a fast learning algorithm, which is widely used in machine learning.

The limited Boltzmann model is given as the sample $X_{I\times L}=\left\{x^{\left(1\right)},x^{\left(2\right)},\cdots ,x^{\left(L\right)}\right\}$ with $x^{\left(l\right)}=\left\{x_{1}^{\left(l\right)},x_{2}^{\left(l\right)},\cdots ,x_{I}^{\left(l\right)}\right\}(1\leq l\leq L)$, which represents training sample l. The weight matrix of the visible and hidden layers of the RBM is *W_J×I_*, and when the sample input is *X_I×L_*, the state of the hidden layer is $H_{J\times L}=W_{J\times I}\times X_{I\times L}$ and can be expressed as follows:

(10) $$H_{J\times L}=W_{J\times I}\times X_{I\times L}=\left[\begin{array}{llll} h_{1}^{\left(1\right)} & h_{1}^{\left(2\right)} & \cdots & h_{1}^{\left(L\right)}\\ h_{2}^{\left(1\right)} & h_{2}^{\left(2\right)} & \cdots & h_{2}^{\left(L\right)}\\ \cdots & \cdots & h_{j}^{\left(l\right)} & \cdots \\ h_{J}^{\left(1\right)} & h_{J}^{\left(2\right)} & \cdots & h_{J}^{\left(L\right)} \end{array}\right]$$

In the formula, each column represents a characteristic corresponding to an input sample, and each row corresponds to the state of one of the hidden units in the hidden layer for all input samples. $h_{j}^{\left(l\right)}$ represents the state of hidden unit *j* when the input is sample *i*.

The traditional standard RBM objective function is as follows:

(11) $$\max_{w,b,c}\sum_{l=1}^{L}\log P(X^{\left(l\right)})$$

*P* is a small constant, and it can be used to control the sparseness, that is, to improve the RBM via sparse filtering.

(12) $$P=s.t.\frac{1}{L}\sum_{l=1}^{L}\hat{h_{j}^{\left(l\right)}},\;\forall j=1,\cdots ,J$$

where $h_{j}^{\left(l\right)}$ is the normalization of each row of the matrix of Formula (12) by *l*_2_.

The re-normalized matrix is now the input. The new matrix now has new row *j* and row *l* elements. In Formula (12), the corresponding constraint on each hidden node is realized. The hidden node is now available to all samples. However, $h_{j}^{\left(l\right)}$ is the result of normalization of the column, so the mean of the square of elements in each column is 1. In the context of different samples, the mean of the activated hidden nodes is basically the same. Because all the hidden nodes in the whole sample have achieved selectivity, only a small part of the hidden nodes is activated. This suggests that sparseness has been achieved.

The process of calculating $h_{j}^{\left(l\right)}$ is as follows:

(1) $l_{2}$ is normalized for each row of matrix (*H_J×L_*), using $h_{j}^{\left(l\right)}$ to denote the value of row *j* and column 1 in the matrix after row normalization. The formula is expressed as follows:
  (13) $$\bar{h_{j}^{\left(l\right)}}=\frac{h_{j}^{\left(l\right)}}{\sum_{l1=1}^{L}h_{j}^{\left(l1\right)}}$$
(2) *l*_2_ is normalized for the columns of the normalized matrix, using $h_{j}^{\left(l\right)}$ to denote the value of row *j* and column 1 in the matrix after row normalization. The formula is expressed as follows:
  (14) $$\hat{h_{j}^{\left(l\right)}}=\frac{\bar{h_{j}^{\left(l\right)}}}{\sum_{j1=1}^{J}\bar{h_{j1}^{\left(l\right)}}}$$

Using this improved RBM in combination with a softmax classifier. The newly developed algorithm is called urban heat island intensity level identification based on RBM-softmax. This classifier will use the hidden layer in an RBM as the input layer of the softmax classifier for the classification.

### 3.5. Construction of the Urban Heat Island Intensity Level Identification Algorithm Based on the Improved RBM

The construction progress of the urban heat island intensity level identification algorithm, based on the improved RBM, is as follows:

(1) A small amount of sample S1 is extracted from the image that has been subjected to the land surface temperature inversion and it is manually marked. The number of artificial markers in a small sample of S1 of each type of clustering center is calculated. At the same time, the unmarked samples (S2) are clustered based on the K-means algorithm.
(2) The Euclidean distance between the two types of cluster centers is compared with that of S1 and S2. Based on the minimum error criterion, the set of assignments with higher confidence, achieved via the K-RBM algorithm, is selected and combined with S1 samples to form the most valuable samples.
(3) For pre-training to improve the RBM, the unsupervised learning of the RBM is improved by using the full sample (S). The next step is carried out when the error between the modified training samples of the RBM and the reconstructed data is quite small. At the same time, the relevant parameters obtained from the pre-training improvement RBM are used in the next step to improve the RBM; otherwise, continue to perform pre-training operations.
(4) The most valuable sample obtained in step 2 is entered into the improved RBM-softmax urban heat island intensity level identification model, training the model parameters by the steepest descent method until convergence.
(5) The data are input into the trained RBM-softmax urban heat intensity level identifier; then, the identification result is obtained, and the classification accuracy is calculated.

**Figure 3:**
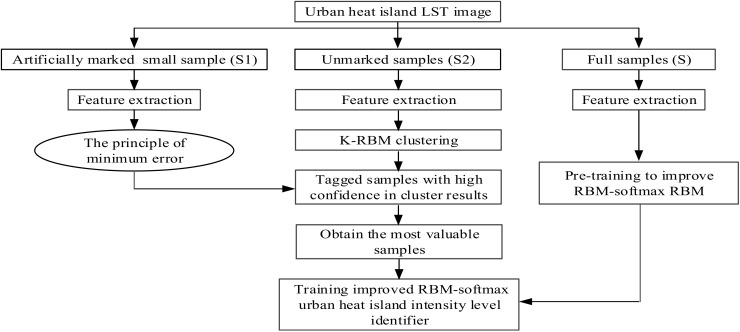
Construction process of the RBM model.

The construction process of the RBM model is shown in Figure 3.

## 4. Results Analysis

### 4.1. Spatial Distribution of Surface Urban Heat Island

**Figure 4:**
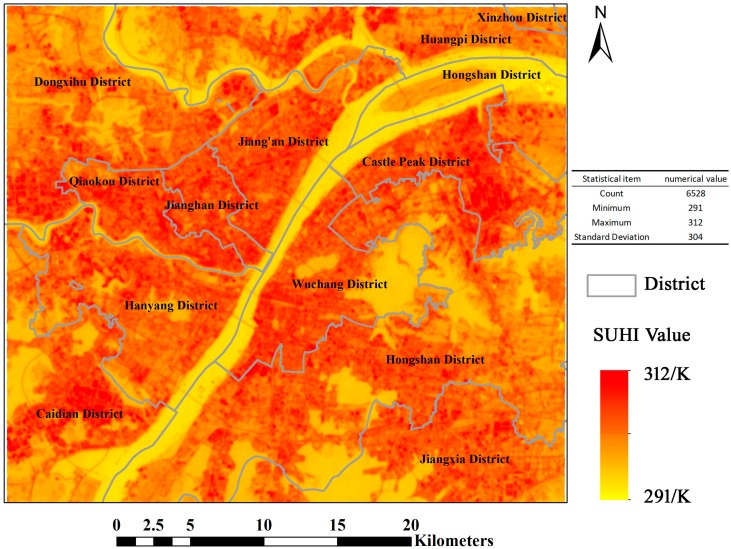
Spatial distribution of the surface urban heat island (SUHI) in Wuhan.

The spatial distribution of the surface urban heat island (SUHI) is drawn, in accordance with the to the land surface temperature inversion method. The Wuhan urban heat island phenomenon is very significant, as shown in Figure 4. High temperatures have an obvious aggregation phenomenon in urban space, but water is a low value area for temperature. The maximum surface temperature is 310 K. This temperature will make people feel very hot.

**Figure 5:**
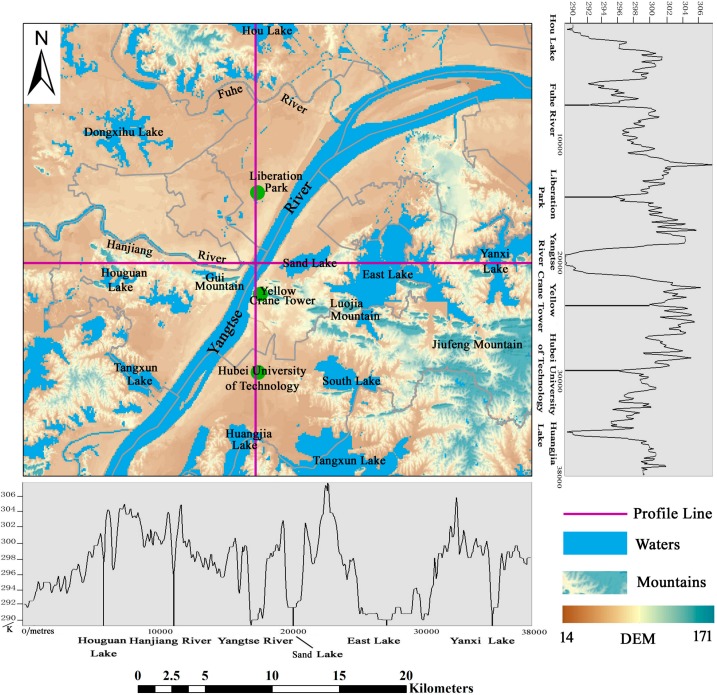
Land surface temperature changes in the profile line.

In order to clearly describe the variation in SUHI temperature, we plotted Figure 5.

The land surface temperature profile for the east–west line in Wuhan, starts from Houguan Lake, through the Hanjiang River, Yangtze River, Sand Lake, East Lake, until Yanxi Lake and its surrounding farmland. In the land surface temperature profile from the east–west in Wuhan, a high, a middle, and a low are shown on both sides of the characteristics. This indicates the existence of urban heat island phenomenon. In addition, the east–west profile has the Yangtze River as its center. The temperature range on both sides of the east–west profile is asymmetric. The main reason for this is that the west area of urban buildings is widely distributed, while the east region of the river area is widely distributed.

From the east–west profile line, the overall urban center temperature is higher than the urban suburbs’ temperatures. The details show a “peak” or “valley” intertwined pattern. The main reason for this is the type of city under the surface. “Valley” mostly appears in farmland, water, and park green space and near the vegetation-covered university.

The north–south profile line, starts from Hou Lake, runs through the Fuhe River, Liberation Park, the Yangtze River, Yellow Crane Tower, Hubei University of Technology, and finally, Huangjia Lake to the surrounding farmland. From the land surface temperature of the north–south profile in Wuhan, a high, a middle, and a low on both sides of the characteristics can also be observed. It can be seen in Figure 5 that the extreme value of the profile line appears in the Jiang’an economic and technological development zone, on the north bank of the river, but the temperature range in the south is higher than that in the north. From the range of changes along the profile line of view, there are still high and low peaks and valleys staggered along the distribution trend. However, the intensity of the north–south profile is not as dramatic as that of the east–west profile.

From the range of the north–south profile line, the waters can form a trough of temperature. Some areas with small building densities and well-covered areas also form large and small cryogenic zones. This includes Hubei University of Technology, Wuhan University campus and other colleges and universities.

### 4.2. Identify the Results

Based on the improved RBM algorithm, an urban heat island intensity level identification map was generated. According to the regional distribution characteristics of the urban heat island and the thermal environment principle, the four categories were named green island, sub-green island, heat island and strong heat island.

The green island, sub-green island, heat island and strong heat island areas in Wuhan are 21,341 hm^2^, 37,753 ha, 50,525 hm^2^ and 19,403 ha. The largest area is that of the heat island level, accounting for 39.19% of the total area of Wuhan, followed by the sub-green island, accounting for 29.28% of the total area of Wuhan. Green island accounts for 16.55% of the total area of Wuhan; the smallest area is that of the strong heat island, accounting for 14.97% of the total area of Wuhan. The total area of the heat island and strong island levels accounted for 54.16% in Wuhan (Table 4); the phenomenon of the heat island effect in Wuhan is obvious, and the heat island areas are widely distributed.

**Figure 6:**
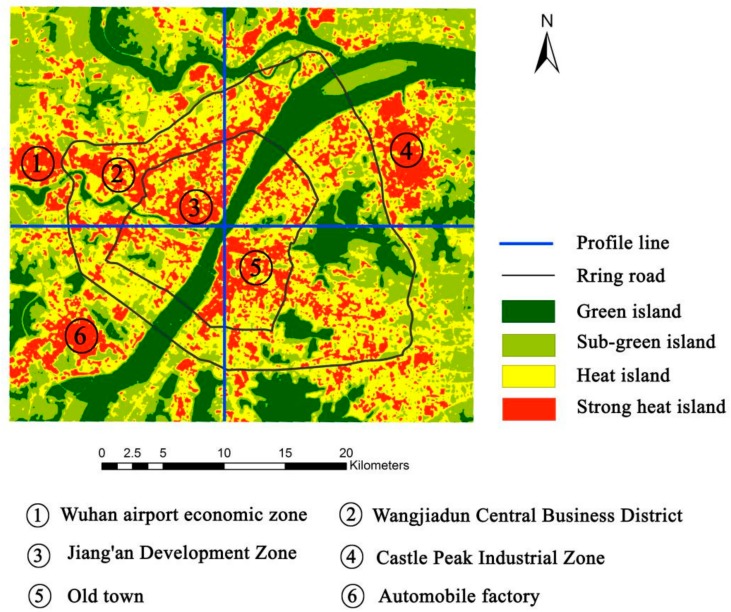
Urban heat island intensity level identification results in Wuhan.

Figure 6 shows that the urban heat island effect in Wuhan is obvious. The main urban areas form an island-like high-temperature area with a clear boundary, which is in sharp contrast to the urban fringe.

The green island is mainly distributed in the Yangtze River, Han River, East Lake, Sand Lake, Tangxun Lake and other water areas of Wuhan. The sub-green island is mainly distributed in Wuhan Jiufeng Mountain, Luojia Mountain, Guishan and other mountains, Yellow Crane Tower, Liberation Park, Evergreen Park and the surrounding areas of farmland and other areas. The hot island is located in the central city of Wuhan and has a large distribution area. The boundary between the heat island and green island, the sub-green island, has a clear line, forming a sharp contrast. The strong heat island is mainly distributed along the Castle Peak Industrial Zone, Wuhan Airport Economic Zone, Jiang’an Development Zone, Wangjiadun Central Business District, the automobile factory area and urban residential intensive areas of Wuhan.

The cause of the heat island effect, in addition to the underlying surface of the media, is the city’s unique heat source conditions, such as plant-based industrial areas, densely populated commercial and living areas, steel mills and power plants. These will only increase and deepen the heat intensity of certain areas.

The surface temperature of industrial-intensive areas was significantly higher than that of other areas in Wuhan. The Castle Peak Industrial Zone is the main distribution area of the strong heat island because it is in the city’s unique heat source zone, where the Wuhan Iron and Steel Plant and Wuhan cooking plants are located. Strong heat islands are mainly distributed in the Jiang’an Development Zone and Wuhan Airport Economic Zone. The Hankou Wangjiadun Central Business District and the old town also form strong heat islands, mainly because these places are in the city where resident activity is intensive.

Regarding the hottest areas that can be seen, most of the “heat” comes from the industrial building areas; secondary heat sources are mainly distributed in dense commercial areas and residential areas. Heavy industry production companies, steel mills and other enterprises in the production industry produce a lot of heat. On the other hand, with the rapid development of the city, the emerging urban areas block the cooling space of these factories and groups, so that the heat island effect in the factory areas is greatly strengthened. At the same time, the vegetation in the factory areas is relatively small, and the solar radiation is strong, which further increases the agglomeration degree of the heat island effect.

From the heat island spatial distribution map, the distribution of water and green space is clearly seen. This distribution of water and green space has a high impact on the urban surface light temperature, which is obvious. Around the several large lakes and urban parks of the main city of Wuhan, there is a green island center in Wuhan. In addition, the large area of water and green areas around some of the urban land also showed a lower temperature, due to an edge effect. The cooling effects of water and vegetation on the urban heat island are of great practical significance for segmenting and controlling the spatial distribution of the heat island within the city.

### 4.3. Comparison of Identification Results

**Figure 7:**
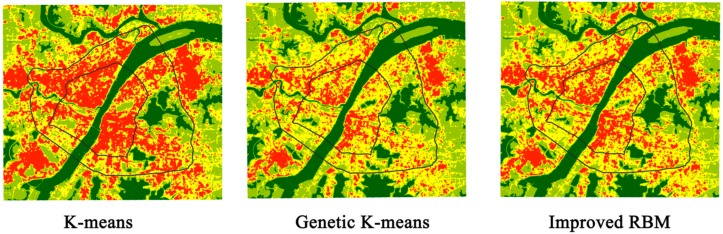
Comparison of identification results obtained by different methods.

The improved RBM identification algorithm was compared with two other models: K-means clustering and genetic K-means clustering [35]. All three models were tested 100 times, and the optimal results were taken. The comparative analysis of three-model is shown in Table 5 and Figure 7, respectively. Under the same experimental conditions, the improved RBM identification algorithm has the highest classification accuracy, with a total accuracy of 93.31% and a Kappa coefficient of 0.8861, which are higher than those for the K-means clustering algorithm and genetic K-means clustering. Among the three algorithms, the improved RBM identification algorithm had the shortest run time at 0.72 s. The genetic K-means clustering algorithm was the second best; however, the K-means clustering had the worst performance. K-means clustering algorithms easily fall into the phenomenon of local optimization because the initial clustering center is more sensitive, so the generated image clustering effect is poor. The differences between the internal temperatures of the city are not obvious, and it cannot achieve the research goals. However, the genetic K-means clustering algorithm does not fall into the local optimum. The algorithm has a good classification effect, and the computational efficiency is higher than that of the K-means clustering algorithm. Compared with the K-means and the genetic K-means, the improved RBM identification algorithm is more obvious in terms of the division of the temperature levels within the city, and the boundary of the categories are obvious. The clustering result of the algorithm can meet the research goal of determining the spatial distribution of the heat island effect within the city. This research method is more practical.

## 5. Discussion

This study reveals that the close relationship between urban heat island intensity and urban land types. In addition, the urban heat island intensity is also strongly correlated with urban morphology. For example, the geometry of different blocks will affect people’s outdoor thermal comfort [36,37]. The research method proposed in this study can also be applied to the study of urban microclimate, which is also a new direction worthy of further study.

The RBM is a variant of the BM (Boltzmann machine, BM), but its limited model must be binary chart. The DBM (Deep Boltzmann machine, DBM) overcomes the problem. The DBM is made up of many RBM stacks. The DBM layers the pre-training RBM with an unsupervised greed pre-training method and the calculation can be made from the initial value of the probability model of supervised learning training. In this way, the learning performance of the whole model can be greatly improved. This will be a continuous exploration direction for this research issue and it can continue to improve the accuracy and computational efficiency of heat island intensity level identification.

## 6. Conclusions

The heat island intensity in an urban space should be a relative temperature concept. It is not an absolute temperature standard that is strictly divided by the absolute value of temperature. The identification model based on the improved RBM heat island strength level was established to solve the problem of subjectivity when dividing the optimal segmentation point of the current heat island. The division points of the heat island intensity under different standards change the urban heat island structure and produce different spatial differentiation rules, which inevitably cause some uncertainty in the research and analysis. Based on the improved RBM model, the urban heat island intensity level identifier is constructed using the idea of weak supervised learning. The identifier automatically initializes the annotation and optimizes the model parameters step-by-step until the target identifier is found. Using very little information about the weak labeling of the target training samples, the urban heat island intensity spatial distribution map is generated. This method also has certain advantages, in comparison with other classification models. Experimental results showed the following:

(1) The largest heat island area is that of the heat island level in Wuhan. Regarding the hottest areas that can be seen, most of the “heat” comes from the industrial building area; secondary heat sources are mainly distributed in dense, commercial areas and residential areas. Heavy industry production companies, steel mills and other enterprises in production projects produce a lot of heat. From the heat island spatial distribution map, the distribution of water and green space can also be clearly seen; the impact of the distribution of water and green space on urban surface light temperature is very obvious. Around the several large lakes and urban parks of the main city of Wuhan, a green island center of Wuhan is formed.
(2) For the RBM identification algorithm, the total accuracy was 93.31%, the kappa coefficient was 0.8861, and the test time was 0.72 s. The algorithm had a good classification effect, and the computational efficiency was higher than that of the K-means clustering algorithm. Compared with K-means and the genetic K-means, the improved RBM identification algorithm is more obvious in terms of the division of the temperature level within the city, and the boundary of the category is obvious. The clustering effect of the algorithm can determine the spatial distribution of the heat island effect within the city, and this method is more practical than others.
(3) Based on the improved RBM model, this paper analyzed the urban heat island and compared it to those obtained with other methods. The focus of the study the spectral characteristics of surface temperature after inversion, which includes texture features for further feature extraction. This method may be enhanced by adding other methods to complete the feature extraction, such as an automatic encoder method. In the process of RBM learning, it is possible to obtain a positive training sample image and a negative training sample image, through the use of a sliding window, to obtain a better distribution rule of the data.

## Figures and Tables

**Table 1:** Acquisition information of remote sensing image data.

Acquisition Time	Line	Number	Image Type	Thermal Infrared Band Spatial Resolution
23 July 2016	123	39	Band 1 Coastal	30 m
			Band 2 Blue	30 m
			Band 3 Green	30 m
			Band 4 Red	30 m
			Band 5 NIR (Near infrared)	30 m
			Band 6 SWIR 1 (Short-wave infrared)	30 m
			Band 7 SWIR 2	30 m
			Band 8 Pan	15 m
			Band 9 Cirrus	30 m
			Band 10 TIRS 1	100 m
			Band 11 TIRS 2	100 m

**Table 2:** Inversion regression coefficients of TIRS in different temperature ranges ($r_{10}^{2}$ and $r_{11}^{2}$ are the determinants of the fit).

Temperature Range/°C	*a*_10_	*b*_10_	$r_{10}^{2}$	*a*_11_	*b*_11_	$r_{11}^{2}$
0–30	−59.139	0.421	0.9991	−63.392	0.457	0.9991
0–40	−60.919	0.428	0.9985	−65.224	0.463	0.9985
10–40	−62.806	0.434	0.9992	−67.173	0.47	0.9992
10–50	−64.608	0.44	0.9986	−69.022	0.476	0.9986

**Table 3:** Relationships between atmospheric transmittance (τ) and water vapor (ω) content when the water vapor content is from 0.5 to 3 g/cm^2^.

Atmospheric Mode	Atmospheric Transmittance Estimation Equation	*r*^2^
The United States in 1976 standard atmosphere	τ10=−0.1146ω+1.0286 τ11=−0.1568ω+1.0083	0.9982 0.9947
Mid-latitude summer	τ10=−0.1134ω+1.0335 τ11=−0.1546ω+1.0078	0.9986 0.9996

**Table 4:** Urban heat island level statistical results in Wuhan.

Heat Island Level	Area	Area Ratio
Green island	21,341	16.55%
Sub-green island	37,753	29.28%
Heat island	50,525	39.19%
Strong heat island	19,304	14.97%

**Table 5:** Identification accuracy comparison of different methods.

Identification Model	K-Means Clustering	Genetic K-Means Clustering	Improved RBM Identifier
Total Accuracy	73.39%	91.22%	93.31%
Kappa	0.6725	0.8735	0.8861
Testing time	2.87	0.91	0.72

## Nomenclature

*L_λ_*	Radiation intensity received by the TM remote sensor
DN	Digital number
*ε*	Specific emissivity
*T*	Radiation light temperature
*A*_0_, *A*_1_, *A*_2_	Coefficient of Formula (5)
τ	Atmospheric transmittance
ω	Water vapor
*a_i_*, *b_i_*	Regression coefficients of TIRS
*E*_0_, *E*_1_, *E*_2_, *C*_i_, *D*_i_	Procedure parameter
*k*, *K*	Classification sequence number, number of classifications, respectively
*m*, *n*	Line sequence number of pixels, column sequence number of pixels, respectively
*M*, *N*	Maximum number of lines and columns in pixels
*i*, *I*	Sequence number of *X*^(*l*)^, total number of *X*^(*l*)^, respectively
*l*, *L*	Training sample sequence number, total number of training samples, respectively
*j*, *J*	Sequence number of hidden units, total number of hidden units, respectively
